# Site-specific metastases of gallbladder adenocarcinoma and their prognostic value for survival: a SEER-based study

**DOI:** 10.1186/s12893-021-01068-8

**Published:** 2021-01-23

**Authors:** Yingnan Yang, Zhuolong Tu, Chentao Ye, Huajie Cai, Shouzhang Yang, Xuehai Chen, Jinfu Tu

**Affiliations:** Department of Hepatobiliary Surgery, First Affiliated Hospital of Wenzhou Medical University, Nanbaixiang Street, Ouhai District, Wenzhou, Zhejiang China

**Keywords:** SEER, Metastases, Gallbladder cancer, Gallbladder adenocarcinoma, Prognosis, Surgical treatment

## Abstract

**Background:**

Gallbladder cancer is a rare but highly malignant cancer, which often progresses to a metastatic stage when diagnosed because of its asymptomatic manifestation. In this study, we intended to analyze the prognostic value of metastatic gallbladder adenocarcinoma (GBA) with site-specific metastases.

**Methods:**

Using the Surveillance, Epidemiology, and End Results (SEER) database, GBA patients diagnosed with metastases between 2010 and 2016 were selected to identify the prognosis according to the isolated metastatic sites, including liver, lung, bone, brain and distant lymph nodes (DL). Kaplan–Meier methods were used for survival comparisons and multivariable Cox regression models were constructed to find out independent factors that associated with survival.

**Results:**

Data from 1526 eligible patients were extracted from the SEER database. Among the patients, 788 (51.6%) had isolated liver metastases, 80 (5.2%) had isolated distant nodal involvement, 45 (2.9%) had isolated lung metastases, 21 (1.4%) had isolated bone metastases, 2 (0.1%) had isolated brain metastases and 590 (38.7%) had multiple metastases. No significant survival difference was shown between patients with single or multisite metastases (P > 0.05). Patients with isolated lung or DL metastases had significant better survival outcomes than those with isolated bone metastases (P < 0.05). Multivariate analysis showed that performing surgery at primary site, receiving chemotherapy were associated with better OS and CSS for patients with isolated liver or DL metastases.

**Conclusions:**

The study showed that different metastatic sites affect survival outcomes in metastatic GBA patients. Highly selected subset of patients with liver or DL metastases might benefit from surgery at primary site.

## Background

Gallbladder cancer (GBC) is a rare gastrointestinal malignancy with an incidence of 1.13/100,000 [1], but it is the most common cancer in the biliary tract [2] and has a dismal prognosis; the 5-year overall survival rate is only 6.7% at stage IVB [3]. Gallbladder adenocarcinoma (GBA) represents the main histological type of GBCs (approximately 76–90%) [4].

The treatment decisions for GBA differ by stage. For T1a tumors, simple cholecystectomy is curative in over 90% of cases [5], and for T1b and more advanced GBA (stage II, III), radical surgery, including lymph node (LN) dissection, should be considered [5, 6]. However, the efficacy of radical surgery and adjuvant therapy for stage IV GBA remains controversial [7]. Some may recommend palliative resection (cholecystectomy with biliary drainage) for stage IV GBA patients [8]. With the increasing incidence of late-stage GBA [9], there are growing number of studies concentrating on its management and prognosis [10–12], however, comprehensive evaluation on the prognostic value of site-specific metastases is lacked. In this article, we aimed to describe the distant metastatic patterns, frequency of occurrence, clinical prognosis of metastatic GBA patients and whether surgical treatment is effective using population-based data from Surveillance, Epidemiology, and End Results (SEER) database.

## Methods

### Data collection

**Fig. 1:**
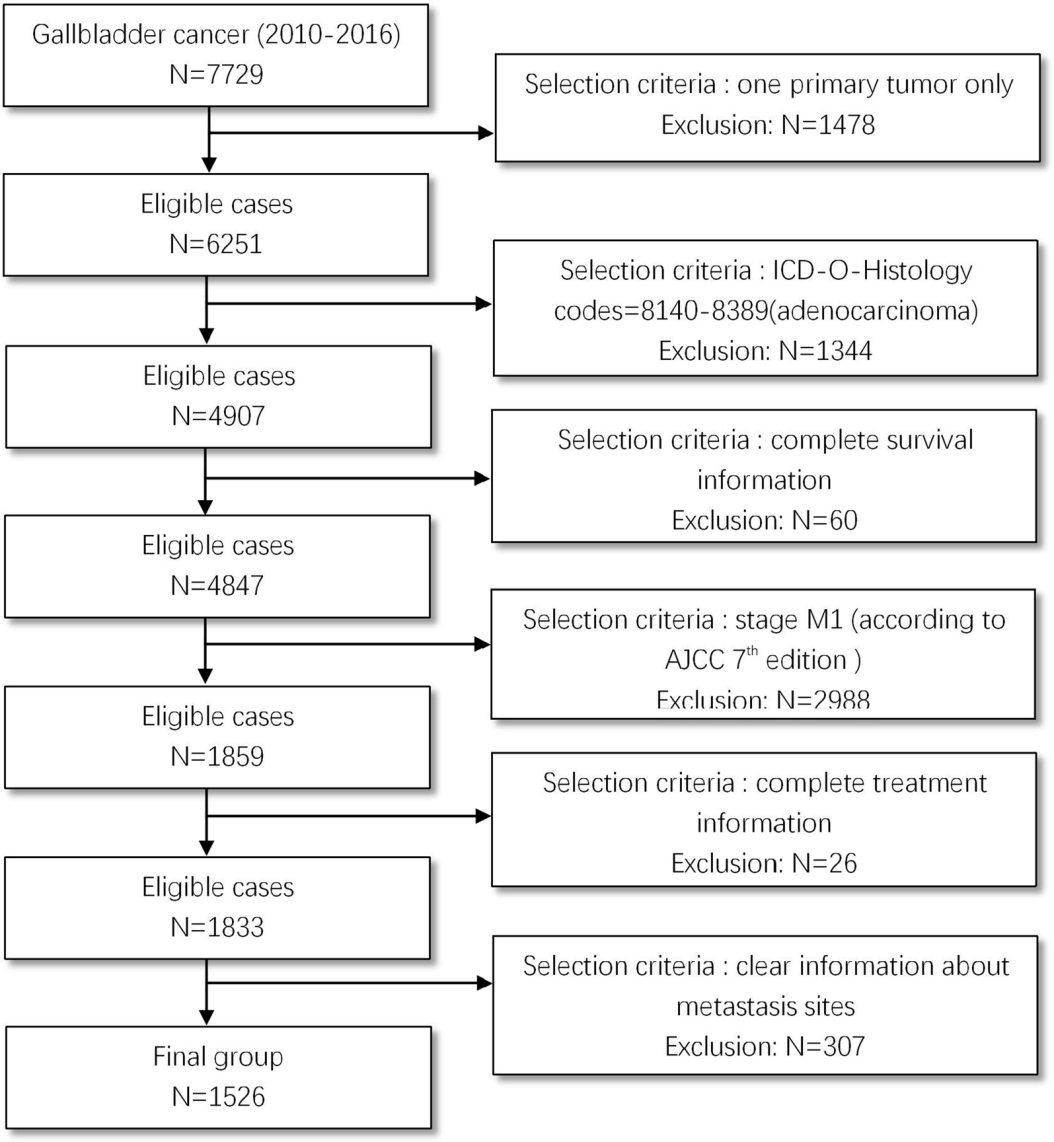
Patient selection flowchart

We extracted data from the SEER database between 2010 and 2016 because information about the metastasis sites was only available beginning in 2010. To identify GBA patients, we selected 7729 cases of “gallbladder” identified by topography code C23.0 (primary gallbladder cancer). Then, the inclusion criteria for metastatic GBA patients were as follows: only one primary site, ICD-O-Histology codes of 8140-8389 (adenocarcinoma), complete survival data, patients who were at AJCC 7th stage M1, complete treatment information (receiving radiation after surgery or not having radiation therapy, receiving surgery or not, receiving chemotherapy or not), having clear metastasis information. All selected patients were older than 18 years old. Detailed selection criteria are show in Fig. 1. Finally, 1526 stage IVB (any T, any N, M1) GBA patients were selected for inclusion in the cohort.

The metastasis information was classified into liver only, lung only, brain only, bone only, distant lymph node (DL) only, and multi-metastasis based on SEER combined mets at DX-liver (2010+), mets at DX-lung (2010+), mets at DX-brain (2010+), mets at DX-bone (2010+), CS mets at DX (2004–2015) and Mets at DX-Distant LN (2016+). Since all the patients were in M1 stage, liver metastases were exclusive of advanced local extension. DL included peripancreatic lymph nodes (along body and tail of pancreas). Celiac, superior mesenteric, para-aortic and pericaval nodes were defined as regional nodes which were not counted as DL in SEER database.

In the SEER database, GBA patients had information about whether surgery was performed at the primary site including simple cholecystectomy with or without hepatectomy based on SEER code for RX Summ-Surg Prim Site (1998+).

Each case includes information on the age of diagnosis, sex, grade, gender, race, marital status, tumor size, site of metastasis, surgery, T stage, N stage, radiation, chemotherapy, survival time and vital status.

### Statistical analysis

The end point of this study was overall survival (OS) and cancer specific survival (CSS) based on the SEER code for cause of death. OS was defined as the duration from diagnosis to death from any cause, and CSS was defined as the duration from diagnosis to death from GBA. In this study, we separated patients into two groups of single-site and multiple site metastases. Single-site metastasis patients were then divided into four groups according to the site of metastasis (liver, lung, bone, and DL), because of the very small number of brain metastasis patients (n = 2), these patients were excluded from the analyses. Clinicopathological characteristics were compared using the Chi-square test. Survival comparisons were calculated by Kaplan–Meier analysis and were examined by log-rank test. We employed the COX proportional model to carry out univariate and multivariate analyses of the patients, hazards ratios (HR) were reported with 95% CI. A P value < 0.05 (two-sided) was considered statistically significant. Statistical analyses were performed using SPSS Statistics 23.0 (IBM, NY, US).

## Results

### Patient characteristics

**Fig. 2:**
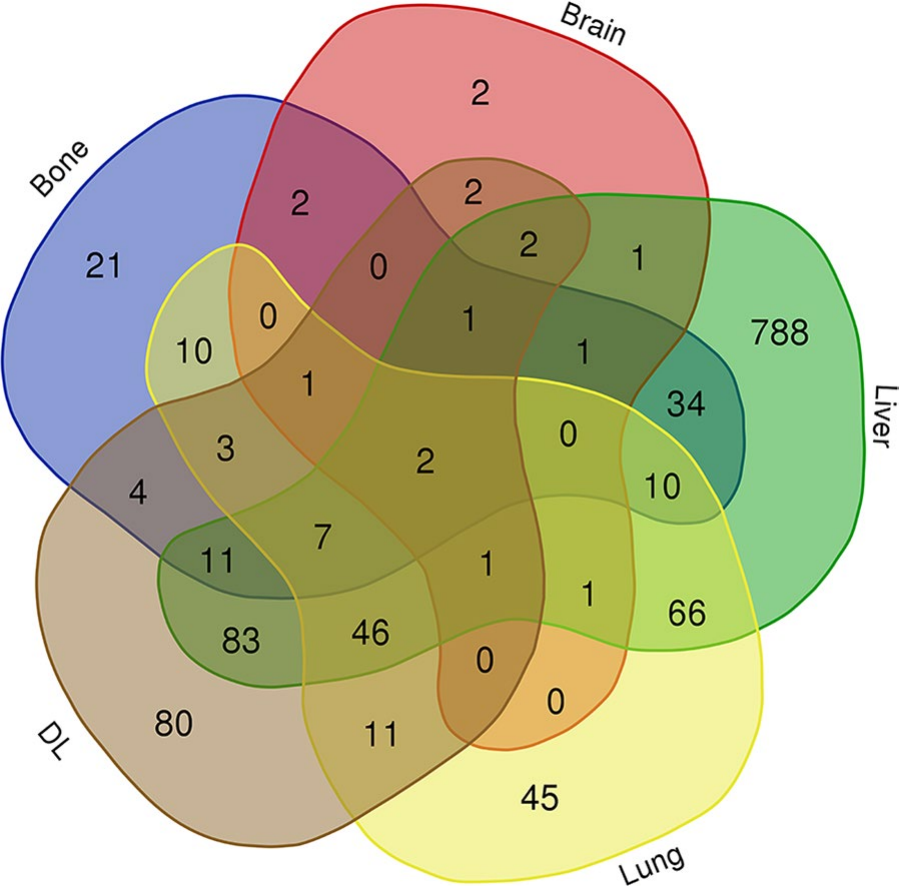
Distant metastasis mode of gallbladder adenocarcinoma

A total of 1526 patients (2010–2016) with known sites of distant metastases were selected for inclusion in this study. A total of 788 (51.6%) patients had isolated liver metastases, 80 (5.2%) patients had isolated DL metastases, 45 (2.9%) patients had isolated lung metastases, 21 (1.4%) patients had isolated bone metastases, 2 (0.1%) patients had isolated brain metastases and 590 (38.7%) patients had multiorgan metastases. The detailed distant metastasis mode was shown in Fig. 2. The mean and median follow-up for the entire cohort were 6.2 and 3 months, respectively. Statistically significant correlations among different baseline characteristics and different metastatic sites from selected GBA patients diagnosed between 2010 and 2016 are summarized in Table 1.

**Table 1:** Clinical features and metastatic sites of metastatic gallbladder adenocarcinoma patients diagnosed between 2010 and 2016

Characteristic	Bone metastases			Brain metastases			Liver metastases			Lung metastases			Distant lymph node metastases		
	Yes	No	P value	Yes	No	P value	Yes	No	P value	Yes	No	P value	Yes	No	P value
Age															
< 60	38 (35.5%)	352 (24.8%)	P = 0.601	9 (56.3%)	381 (25.2%)	P = 0.069	266 (25.2%)	124 (26.3%)	P = 0.543	50 (24.6%)	340 (25.7%)	P = 0.205	79 (31.1%)	311 (24.4%)	P = 0.020
60–69	34 (31.8%)	426 (30.0%)		3 (18.8%)	457 (30.3%)		314 (29.8%)	146 (30.9%)		53 (26.1%)	407 (30.8%)		73 (28.7%)	387 (30.4%)	
70–79	22 (20.6%)	396 (27.9%)		2 (12.5%)	416 (27.5%)		286 (27.1%)	132 (28.0%)		68 (33.5%)	350 (26.5%)		73 (28.7%)	345 (27.1%)	
≥ 80	13 (12.1%)	245 (17.3%)		2 (12.5%)	256 (17.0%)		188 (17.8%)	70 (14.8%)		32 (15.8%)	226 (17.1%)		29 (11.4%)	229 (18.0%)	
Gender															
Male	39 (36.4%)	425 (30.0%)	P = 0.165	7 (43.8%)	457 (30.3%)	P = 0.259	39 (36.4%)	425 (30.0%)	P = 0.954	70 (34.5%)	394 (29.8%)	P = 0.179	90 (35.4%)	374 (29.4%)	P = 0.059
Female	68 (63.6%)	994 (70.0%)		9 (56.3%)	1053 (69.7%)		68 (63.6%)	994 (70.0%)		133 (65.5%)	929 (70.2%)		164 (64.6%)	898 (70.6%)	
Marriage															
Married	67 (62.6%)	751 (52.9%)	P = 0.132	10 (62.5%)	808 (53.5%)	P = 0.601	556 (52.8%)	262 (55.5%)	P = 0.603	104 (51.2%)	714 (54.0%)	P = 0.766	138 (54.3%)	680 (53.5%)	P = 0.923
Unmarried	36 (33.6%)	616 (43.4%)		5 (31.3%)	647 (42.8%)		459 (43.5%)	193 (40.9%)		91 (44.8%)	561 (42.4%)		106 (41.7%)	546 (42.9%)	
Unknown	4 (3.7%)	52 (3.7%)		1 (6.3%)	55 (3.6%)		39 (3.7%)	17 (3.6%)		8 (3.9%)	48 (3.6%)		10 (3.9%)	46 (3.6%)	
Race															
White	82 (76.6%)	1061 (74.8%)	P = 0.731	13 (81.3%)	1130 (74.8%)	P = 0.062	775 (73.5%)	368 (78.0%)	P = 0.172	156 (76.8%)	987 (74.6%)	P = 0.659	185 (72.8%)	958 (75.3%)	P = 0.421
Black	16 (15.0%)	205 (14.4%)		0 (0.0%)	221 (14.6%)		162 (15.4%)	59 (12.5%)		29 (14.3%)	192 (14.5%)		36 (14.2%)	185 (14.5%)	
Others	9 (8.4%)	153 (10.8%)		3 (18.8%)	159 (10.5%)		117 (11.1%)	45 (9.5%)		18 (8.9%)	144 (10.9%)		33 (13.0%)	129 (10.1%)	
Grade															
I	2 (1.9%)	47 (3.3%)	P = 0.107	0 (0.0%)	49 (3.2%)	P = 0.263	23 (2.2%)	26 (5.5%)	P = 0.01	5 (2.5%)	44 (3.3%)	P = 0.236	4 (1.6%)	45 (3.5%)	P = 0.059
II	11 (10.3%)	257 (18.1%)		1 (6.3%)	267 (17.7%)		171 (16.2%)	97 (20.6%)		30 (14.8%)	238 (18.0%)		49 (19.3%)	219 (17.2%)	
III + IV	30 (28.0%)	385 (27.1%)		7 (43.8%)	408 (27.0%)		293 (27.8%)	122 (25.8%)		49 (24.1%)	366 (27.7%)		57 (22.4%)	358 (28.1%)	
Unknown	64 (59.8%)	730 (51.4%)		8 (50.0%)	786 (52.1%)		567 (53.8%)	227 (48.1%)		119 (58.6%)	675 (51.0%)		144 (56.7%)	650 (51.1%)	
T stage															
T1	5 (4.7%)	15 (1.1%)	P = 0.020	2 (12.5%)	18 (1.2%)	P = 0.159	13 (1.2%)	7 (1.5%)	P = 0.007	7 (3.4%)	13 (1.0%)	P = 0.015	9 (3.5%)	11 (0.9%)	P = 0.017
T2	6 (5.6%)	114 (8.0%)		1 (6.3%)	119 (7.9%)		90 (8.5%)	30 (6.4%)		21 (10.3%)	99 (7.5%)		17 (6.7%)	103 (8.1%)	
T3	10 (9.3%)	174 (12.3%)		2 (12.5%)	182 (12.1%)		122 (11.6%)	62 (13.1%)		16 (7.9%)	168 (12.7%)		22 (8.7%)	162 (12.7%)	
T4	43 (40.2%)	643 (45.3%)		4 (25.0%)	682 (45.2%)		453 (43.0%)	233 (49.4%)		89 (43.8%)	597 (45.1%)		117 (46.1%)	569 (44.7%)	
TX	4 (3.7%)	97 (6.8%)		1 (6.3%)	100 (6.6%)		63 (6.0%)	38 (8.1%)		9 (4.4%)	92 (7.0%)		21 (8.3%)	80 (6.3%)	
Unknown	39 (36.4%)	376 (26.5%)		6 (37.5%)	409 (27.1%)		313 (29.7%)	102 (21.6%)		61 (30.0%)	354 (26.8%)		68 (26.8%)	347 (27.3%)	
N stage															
N0	49 (45.8%)	635 (44.7%)	P = 0.0295	4 (25.0%)	680 (45.0%)	P = 0.272	443 (42.0%)	241 (51.1%)	P = 0.002	77 (37.9%)	607 (45.9%)	P = 0.068	53 (20.9%)	631 (49.6%)	P < 0.001
N1	21 (19.6%)	385 (27.1%)		4 (25.0%)	402 (26.6%)		303 (28.7%)	103 (21.8%)		69 (34.0%)	337 (25.5%)		84 (33.1%)	322 (25.3%)	
N2	17 (15.9%)	180 (12.7%)		4 (25.0%)	193 (12.8%)		132 (12.5%)	65 (13.8%)		25 (12.3%)	172 (13.0%)		88 (34.6%)	109 (8.6%)	
NX	20 (18.7%)	219 (15.4%)		4 (25.0%)	235 (15.6%)		176 (16.7%)	63 (13.3%)		32 (15.8%)	207 (15.6%)		29 (11.4%)	210 (16.5%)	
Surgery at primary site															
Yes	26 (24.3%)	504 (35.5%)	P = 0.016	4 (25.0%)	526 (34.8%)	P = 0.399	337 (32.0%)	193 (40.9%)	P = 0.001	38 (18.7%)	492 (37.2%)	P < 0.001	57 (22.4%)	473 (37.2%)	P < 0.001
No	81 (75.7%)	915 (64.5%)		12 (75.0%)	984 (65.2%)		717 (68.0%)	279 (59.1%)		165 (81.3%)	831 (62.8%)		197 (77.6%)	799 (62.8%)	
Radiation															
Yes	31 (29.0%)	78 (5.5%)	P < 0.001	11 (68.8%)	98 (6.5%)	P < 0.001	67 (6.4%)	42 (8.9%)	P = 0.080	16 (7.9%)	93 (7.0%)	P = 0.665	25 (9.8%)	84 (6.6%)	P = 0.079
No	76 (71.0%)	1341 (94.5%)		5 (31.3%)	1412 (93.5%)		987 (93.6%)	430 (91.1%)		187 (92.1%)	1230 (93.0%)		229 (90.2%)	1188 (93.4%)	
Chemotherapy															
Yes	58 (54.2%)	750 (52.9%)	P = 0.787	10 (62.5%)	798 (52.8%)	P = 0.438	538 (51.0%)	270 (57.2%)	P = 0.026	111 (54.7%)	697 (52.7%)	P = 0.596	161 (63.4%)	647 (50.9%)	P < 0.001
No	49 (45.8%)	669 (47.1%)		6 (37.5%)	712 (47.2%)		516 (49.0%)	202 (42.8%)		92 (45.3%)	626 (47.3%)		93 (36.6%)	625 (49.1%)	
Tumor size															
< 3	11 (10.3%)	201 (14.2%)	P = 0.167	3 (18.8%)	209 (13.8%)	P = 0.183	132 (12.5%)	80 (16.9%)	P = 0.122	30 (14.8%)	182 (13.8%)	P = 0.046	35 (13.8%)	177 (13.9%)	P = 0.998
< 5	12 (11.2%)	197(13.9%)		0 (0.0%)	209 (13.8%)		142 (13.5%)	67 (14.2%)		22 (10.8%)	187 (14.1%)		34 (13.4%)	175 (13.8%)	
≥ 5	15 (14.0%)	263 (18.5%)		3 (18.8%)	275 (18.2%)		195 (18.5%)	83 (17.6%)		26 (12.8%)	252 (19.0%)		47 (18.5%)	231 (18.2%)	
Unknown	69 (64.5%)	758 (53.4%)		10 (62.5%)	817 (54.1%)		585 (55.5%)	242 (51.3%)		125 (61.6%)	702 (53.1%)		138 (54.3%)	689 (54.2%)	

Other races include Asian or Pacific Islander and American Indian/Alaska Native; Grade I = well differentiated, II = moderately differentiated, III = poorly differentiated, IV = undifferentiated

### Survival outcomes

**Fig. 3:**
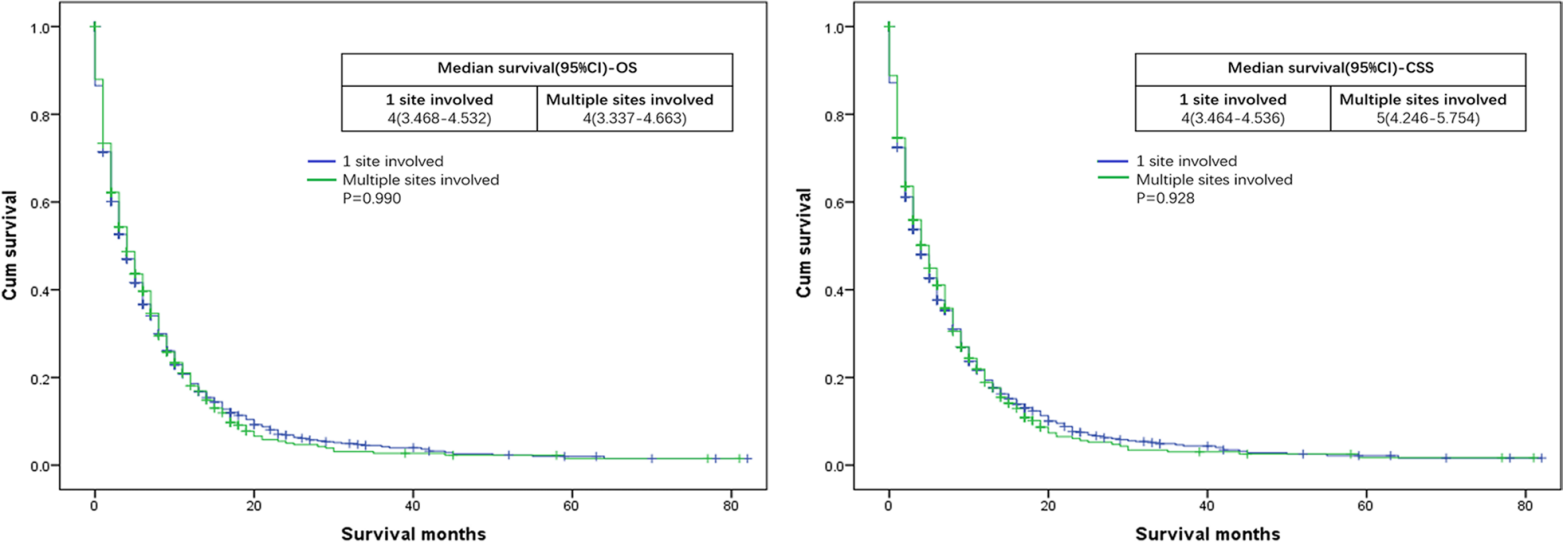
Kaplan–Meier curves of overall survival (OS) and cancer specific survival (CSS) according to the number of involved sites

OS and CSS were compared according to the site of metastasis. Median OS for single and multiple metastatic GBA patients both were 4 months. Median CSS for single and multiple metastatic GBA patients were 4 and 5 months, respectively (for OS: P = 0.990; for CSS: P = 0.928) (Fig. 3).

**Fig. 4:**
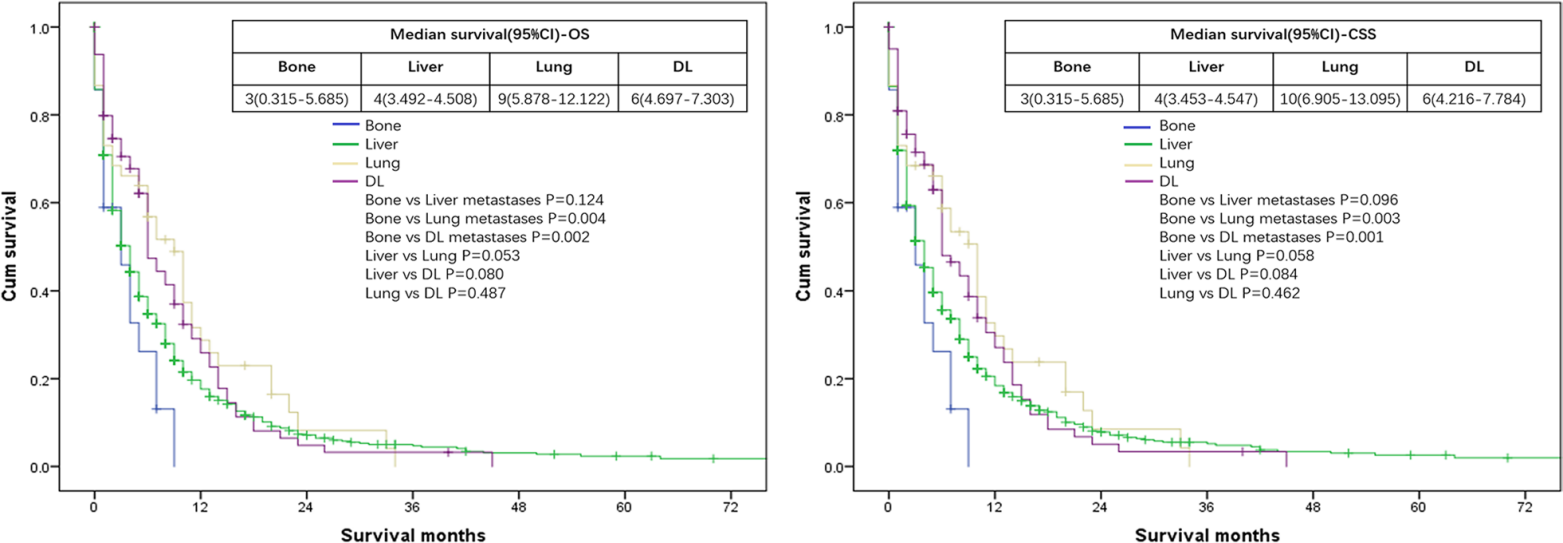
Kaplan–Meier curves of overall survival (OS) and cancer specific survival (CSS) according to the isolated site of metastases

Intergroup analysis showed that respective OS and CSS were both 3 months for patients with isolated bone metastases, both 4 months for patients with isolated liver metastases, 9 and 10 months for patients with isolated lung metastases, both 6 months for patients with isolated DL metastases. Both end points showed that patients with isolated lung or DL metastases had better survival outcomes compared with patients with isolated bone metastases (for OS: DL vs bone metastases: P = 0.002; lung vs bone metastases: P = 0.004) (for CSS: DL vs bone metastases: P = 0.001; lung vs bone metastases: P = 0.003) (Fig. 4).

**Fig. 5:**
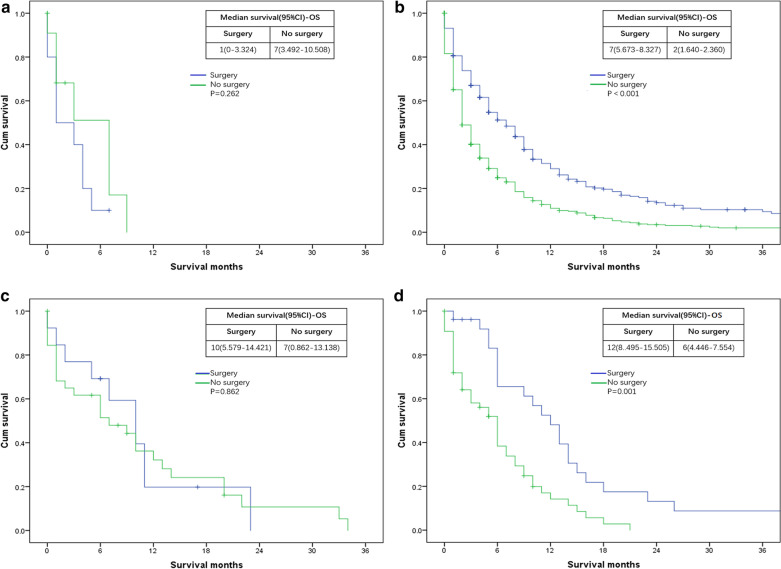
Kaplan–Meier curves of overall survival (OS) according to whether or not surgery at the primary lesion has been done. **a** patients with bone metastases only: surgery = 10 patients, no surgery = 11 patients; **b** patients with liver metastases only: surgery = 290 patients, no surgery = 498 patients; **c** patients with lung metastases only: surgery = 13 patients, no surgery = 32 patients; **d** patients with DL metastases only: surgery = 26 patients, no surgery = 54 patients

**Fig. 6:**
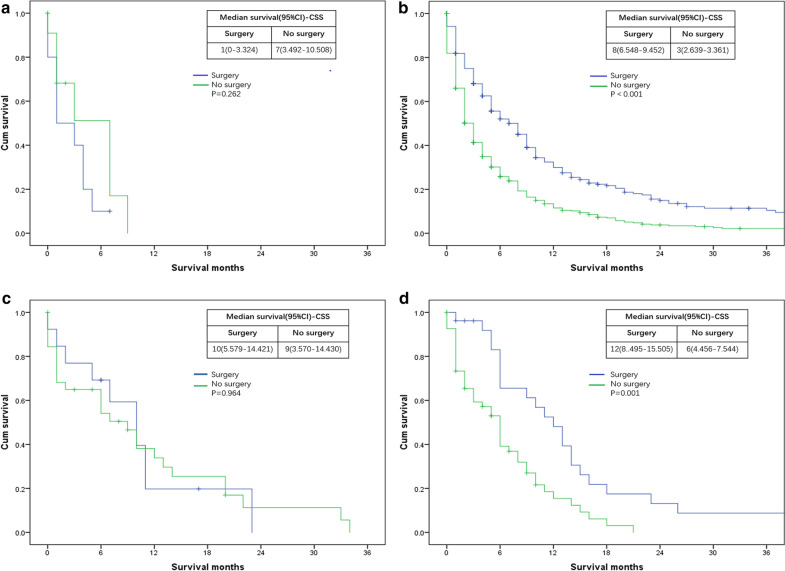
Kaplan–Meier curves of cancer specific survival (CSS) according to whether or not surgery at the primary lesion has been done. **a** patients with bone metastases only: surgery = 10 patients, no surgery = 11 patients; **b** patients with liver metastases only: surgery = 290 patients, no surgery = 498 patients; **c** patients with lung metastases only: surgery = 13 patients, no surgery = 32 patients; **d** patients with DL metastases only: surgery = 26 patients, no surgery = 54 patients

Analysis was also evaluated according to whether or not perform surgery at primary site. We found that surgery at the primary site resulted in no statistically significant survival difference compared to patients who did not receive surgery with bone (for OS: P = 0.262; for CSS: P = 0.262) (Figs. 5a, 6a) and lung (for OS: P = 0.862; for CSS: P = 0.964) (Figs. 5c, 6c) metastases. However, surgery at primary site were significantly beneficial for patients with liver (for OS: P < 0.01; for CSS: P < 0.01) (Figs. 5b, 6b) or DL (for OS: P = 0.01; for CSS: P = 0.01) (Figs. 5d, 6d) metastases compared to those not undergoing surgery.

### Multivariable Cox regression models

We conducted univariate analysis on each factor, and brought statistically significant factors into the multivariate model. For the entire cohort, smaller tumor size, high histological grade, performing surgery at primary site, receiving chemotherapy were associated with better OS and CSS (Table 2).

**Table 2:** Univariate and multivariate COX regression analyses for metastatic gallbladder adenocarcinoma patients

Features	Overall survival				Cancer specific survival			
	Univariate		Multivariate		Univariate		Multivariate	
	HR (95% CI)	P-value	HR (95% CI)	P-value	HR (95% CI)	P-value	HR (95% CI)	P-value
Age								
< 60	1.000 (Reference)		1.000 (Reference)		1.000 (Reference)		1.000 (Reference)	
60–69	1.045 (0.900–1.212)	0.566	1.029 (0.885–1.195)	0.711	1.023 (0.880–1.190)	0.764	1.012 (0.869–1.178)	0.879
70–79	1.243 (1.070–1.444)	0.004	1.042 (0.894–1.215)	0.599	1.221 (1.049–1.421)	0.010	1.027 (0.879–1.200)	0.736
≥ 80	1.774 (1.499–2.100)	< 0.001	1.167 (0.975–1.396)	0.092	1.758 (1.482–2.085)	0.000	1.156 (0.964–1.387)	0.117
Gender								
Male	1.000 (Reference)		1.000 (Reference)		1.000 (Reference)			
Female	0.878 (0.779–0.988)	0.031	0.914 (0.809–1.033)	0.151	0.897 (0.794–1.012)	0.077		
Marriage								
Married	1.000 (Reference)		1.000 (Reference)		1.000 (Reference)		1.000 (Reference)	
Unmarried	1.204 (1.076–1.346)	0.001	1.026 (0.910–1.155)	0.679	1.206 (1.0761.352)	0.001	1.012 (0.898–1.140)	0.844
Unknown	1.166 (0.870–1.563)	0.304	0.874 (0.647–1.180)	0.380	1.180 (0.878–1.587)	0.273	0.880 (0.650–1.192)	0.411
Race								
White	1.000 (Reference)				1.000 (Reference)			
Black	1.033 (0.883–1.209)	0.683			1.031 (0.879–1.208)	0.711		
Others	1.050 (0.879–1.254)	0.589			1.043 (0.871–1.250)	0.645		
Grade								
I	1.000 (Reference)		1.000 (Reference)		1.000 (Reference)		1.000 (Reference)	
II	1.087 (0.770–1.535)	0.636	1.173 (0.828–1.662)	0.368	1.046 (0.740–1.478)	0.800	1.119 (0.789–1.587)	0.527
III + IV	1.643 (1.174–2.298)	0.004	1.704 (1.212–2.397)	0.002	1.582 (1.130–2.214)	0.008	1.639 (1.165–2.307)	0.005
Unknown	1.893 (1.364–2.627)	< 0.001	1.489 (1.046–2.120)	0.027	1.845 (1.329–2.560)	< 0.001	1.455 (1.021–2.073)	0.038
T stage								
T0	1.000 (Reference)		1.000 (Reference)		1.000 (Reference)		1.000 (Reference)	
T1	1.048 (0.644–1.704)	0.851	1.349 (0.823–2.211)	0.235	1.009 (0.620–1.643)	0.971	1.273 (0.776–2.088)	0.339
T2	0.590 (0.365–0.952)	0.031	1.027 (0.621–1.699)	0.917	0.568 (0.351–0.918)	0.021	0.982 (0.593–1.626)	0.943
T3	0.877 (0.555–1.386)	0.574	1.082 (0.678–1.725)	0.741	0.849 (0.537–1.341)	0.483	1.034 (0.648–1.649)	0.889
T4	0.869 (0.528–1.431)	0.581	0.948 (0.573–1.568)	0.835	0.858 (0.521–1.414)	0.548	0.926 (0.560–1.532)	0.764
TX	1.087 (0.685–1.725)	0.725	1.077 (0.677–1.714)	0.755	1.048 (0.660–1.664)	0.843	1.027 (0.645–1.635)	0.911
N stage								
N0	1.000 (Reference)				1.000 (Reference)			
N1	0.937 (0.820–1.070)	0.336			0.938 (0.819–1.074)	0.352		
N2	0.985 (0.827–1.173)	0.863			1.005 (0.842–1.198)	0.958		
NX	1.359 (1.156–1.597)	< 0.001			1.352 (1.147–1.594)	< 0.001		
Surgery at primary site								
Yes	1.000 (Reference)		1.000 (Reference)		1.000 (Reference)		1.000 (Reference)	
No	1.633 (1.452–1.836)	< 0.001	1.578 (1.329–1.873)	< 0.001	1.641 (1.456–1.849)	< 0.001	1.588 (1.334–1.890)	< 0.001
Radiation								
Yes	1.000 (Reference)		1.000 (Reference)		1.000 (Reference)		1.000 (Reference)	
No	1.482 (1.188–1.849)	< 0.001	1.125 (0.8981.409)	0.307	1.454 (1.163–1.817)	0.001	1.107 (0.882–1.388)	0.380
Chemotherapy								
Yes	1.000 (Reference)		1.000 (Reference)		1.000 (Reference)		1.000 (Reference)	
No	2.554 (2.282–2.858)	< 0.001	2.711 (2.3973.066)	< 0.001	2.556 (2.280–2.865)	< 0.001	2.723 (2.404–3.086)	< 0.001
Metastatic type								
Single site	1.000 (Reference)				1.000 (Reference)			
Multiple sites	1.001 (0.894–1.120)	0.990		0.933	0.995 (0.888–1.116)	0.933		
Tumor size								
< 3 cm	1.000 (Reference)		1.000 (Reference)		1.000 (Reference)		1.000 (Reference)	
< 5 cm	1.373 (1.109–1.700)	0.004	1.340 (1.076–1.668)	0.009	1.372 (1.106–1.703)	0.004	1.330 (1.066–1.659)	0.012
≥ 5 cm	1.420 (1.164–1.731)	0.001	1.354 (1.101–1.666)	0.004	1.391 (1.138–1.701)	0.001	1.327 (1.076–1.636)	0.008
Unknown	1.657 (1.399–1.961)	< 0.001	1.387 (1.158–1.661)	< 0.001	1.630 (1.374–1.933)	< 0.001	1.356 (1.130–1.627)	0.001

Other races include Asian or Pacific Islander and American Indian/Alaska Native; Grade I = well differentiated, II = moderately differentiated, III = poorly differentiated, IV = undifferentiated; HR = hazard ratio; CI = confidence interval

We then performed multivariate analyses of patients with isolated liver and DL metastases using the same method to further explore the independent prognostic factors on the OS and CSS, and the results suggested that for patients with isolated liver metastases, performing surgery at primary site, receiving chemotherapy were associated with better OS and CSS. Grade I was only related to better OS (Table 3). For patients with isolated DL metastases, performing surgery at primary site, receiving chemotherapy were associated with better OS and CSS (Table 4).

**Table 3:** Univariate and multivariate COX regression analyses for patients with isolated liver metastases

Features	Overall survival				Cancer specific survival			
	Univariate		Multivariate		Univariate		Multivariate	
	HR (95% CI)	P-value	HR (95% CI)	P-value	HR (95% CI)	P-value	HR (95% CI)	P-value
Age								
< 60	1.000 (Reference)		1.000 (Reference)		1.000 (Reference)		1.000 (Reference)	
60–69	1.136 (0.920–1.402)	0.236	1.171 (0.944–1.452)	0.151	1.115 (0.900–1.382)	0.318	1.149 (0.923–1.430)	0.214
70–79	1.313 (1.062–1.623)	0.012	1.155 (0.929–1.436)	0.194	1.325 (1.069–1.641)	0.010	1.165 (0.934–1.451)	0.175
≥ 80	1.990 (1.580–2.505)	< 0.001	1.233 (0.960–1.583)	0.101	1.966 (1.556–2.485)	< 0.001	1.216 (0.944–1.567)	0.131
Gender								
Male	1.000 (Reference)				1.000 (Reference)			
Female	0.914 (0.775–1.078)	0.286			0.934 (0.789–1.105)	0.425		
Marriage								
Married	1.000 (Reference)		1.000 (Reference)		1.000 (Reference)		1.000 (Reference)	
Unmarried	1.245 (1.066–1.455)	0.006	1.006 (0.853–1.186)	0.945	1.243 (1.061–1.455)	0.007	1.003 (0.849–1.186)	0.969
Unknown	1.119 (0.751–1.668)	0.579	1.057 (0.699–1.600)	0.792	1.109 (0.738–1.664)	0.619	1.039 (0.681–1.585)	0.858
Race								
White	1.000 (Reference)				1.000 (Reference)			
Black	0.995 (0.804–1.232)	0.964			0.993 (0.800–1.233)	0.953		
Others	0.939 (0.730–1.208)	0.624			0.924 (0.714–1.195)	0.545		
Grade								
I	1.000 (Reference)		1.000 (Reference)		1.000 (Reference)		1.000 (Reference)	
II	1.240 (0.697–2.209)	0.464	1.087 (0.604–1.957)	0.781	1.202 (0.674–2.143)	0.532	1.047 (0.581–1.887)	0.879
III + IV	1.982 (1.129–3.479)	0.017	1.829 (1.035–3.233)	0.038	1.917 (1.091–3.367)	0.024	1.745 (0.986–3.088)	0.056
Unknown	2.396 (1.376–4.174)	0.002	1.553 (0.861–2.800)	0.143	2.324 (1.334–4.050)	0.003	1.478 (0.818–2.669)	0.195
T stage								
T0	1.000 (Reference)		1.000 (Reference)		1.000 (Reference)		1.000 (Reference)	
T1	0.630 (0.288–1.376)	0.247	0.683 (0.309–1.512)	0.347	0.611 (0.279–1.337)	0.217	0.666 (0.301–1.477)	0.317
T2	0.374 (0.172–0.811)	0.013	0.664 (0.296–1.490)	0.321	0.364 (0.168–0.792)	0.011	0.669 (0.298–1.503)	0.331
T3	0.551 (0.260–1.168)	0.120	0.666 (0.308–1.437)	0.300	0.540 (0.255–1.145)	0.108	0.659 (0.305–1.424)	0.289
T4	0.573 (0.256–1.283)	0.176	0.509 (0.224–1.153)	0.105	0.574 (0.256–1.284)	0.177	0.510 (0.225–1.157)	0.107
TX	0.713 (0.335–1.517)	0.380	0.594 (0.277–1.274)	0.181	0.678 (0.319–1.443)	0.313	0.564 (0.263–1.210)	0.142
N stage								
N0	1.000 (Reference)				1.000 (Reference)			
N1	0.907 (0.755–1.090)	0.299			0.916 (0.760–1.103)	0.353		
N2	1.102 (0.844–1.438)	0.474			1.142 (0.874–1.492)	0.329		
NX	1.356 (1.094–1.682)	0.005			1.359 (1.092–1.691)	0.006		
Surgery at primary site								
Yes	1.000 (Reference)		1.000 (Reference)		1.000 (Reference)		1.000 (Reference)	
No	1.797 (1.528–2.112)	< 0.001	1.800 (1.401–2.313)	< 0.001	1.827 (1.550–2.154)	< 0.001	1.901 (1.474–2.453)	< 0.001
Radiation								
Yes	1.000 (Reference)		1.000 (Reference)		1.000 (Reference)		1.000 (Reference)	
No	1.879 (1.302–2.712)	0.001	1.258 (0.861–1.838)	0.236	1.8211.261–2.629)	0.001	1.230 (0.841–1.798)	0.286
Chemotherapy								
Yes	1.000 (Reference)		1.000 (Reference)		1.000 (Reference)		1.000 (Reference)	
No	2.600 (2.222–3.042)	< 0.001	2.700 (2.263–3.221)	< 0.001	2.599 (2.216–3.048)	< 0.001	2.723 (2.276–3.258)	< 0.001
Tumor size								
< 3 cm	1.000 (Reference)		1.000 (Reference)		1.000 (Reference)		1.000 (Reference)	
< 5 cm	1.414 (1.051–1.902)	0.022	1.326 (0.979–1.797)	0.069	1.417 (1.050–1.913)	0.023	1.321 (0.971–1.797)	0.076
≥ 5 cm	1.349 (1.021–1.783)	0.035	1.269 (0.946–1.702)	0.112	1.372 (1.036–1.818)	0.027	1.279 (0.950–1.720)	0.104
Unknown	1.650 (1.300–2.095)	< 0.001	1.392 (1.079–1.795)	0.011	1.623 (1.274–2.068)	0.000	1.361 (1.051–1.762)	0.019

Other races include Asian or Pacific Islander and American Indian/Alaska Native; Grade I = well differentiated, II = moderately differentiated, III = poorly differentiated, IV = undifferentiated; HR = hazard ratio; CI = confidence interval

**Table 4:** Univariate and multivariate COX regression analyses for patients with isolated distant lymph node metastases

Features	Overall survival				Cancer specific survival			
	Univariate		Multivariate		Univariate		Multivariate	
	HR (95% CI)	P-value	HR (95% CI)	P-value	HR (95% CI)	P-value	HR (95% CI)	P-value
Age								
< 60	1.000 (Reference)				1.000 (Reference)			
60–69	0.761 (0.393–1.472)	0.416			0.755 (0.390–1.463)	0.405		
70–79	1.054 (0.554–2.006)	0.872			0.957 (0.497–1.845)	0.897		
≥ 80	1.695 (0.691–4.160)	0.249			1.726 (0.703–4.239)	0.234		
Gender								
Male	1.000 (Reference)				1.000 (Reference)			
Female	0.747 (0.453–1.232)	0.254			0.710 (0.429–1.177)	0.184		
Marriage								
Married	1.000 (Reference)				1.000 (Reference)			
Unmarried	0.615 (0.363–1.042)	0.071			0.645 (0.379–1.097)	0.106		
Unknown	1.133 (0.348–3.691)	0.836			1.208 (0.370–3.944)	0.755		
Race								
White	1.000 (Reference)				1.000 (Reference)			
Black	0.491 (0.225–1.071)	0.074			0.510 (0.233–1.115)	0.091		
Others	0.741 (0.377–1.457)	0.385			0.770 (0.391–1.518)	0.450		
Grade								
I	1.000 (Reference)				1.000 (Reference)			
II	0.466 (0.102–2.132)	0.325			0.466 (0.102–2.133)	0.325		
III + IV	1.133 (0.257–4.985)	0.869			1.061 (0.240–4.700)	0.938		
Unknown	1.465 (0.350–6.129)	0.601			1.436 (0.343–6.015)	0.621		
T stage								
T0	1.000 (Reference)				1.000 (Reference)			
T1	0.513 (0.114–2.309)	0.385			0.510 (0.113–2.297)	0.381		
T2	0.340 (0.087–1.328)	0.121			0.330 (0.084–1.295)	0.112		
T3	0.483 (0.145–1.612)	0.237			0.441 (0.132–1.481)	0.185		
T4	0.788 (0.202–3.070)	0.731			0.785 (0.201–3.062)	0.728		
TX	0.946 (0.272–3.286)	0.930			0.943 (0.271–3.278)	0.926		
N stage								
N0	1.000 (Reference)				1.000 (Reference)			
N1	0.623 (0.315–1.229)	0.172			0.610 (0.303–1.229)	0.167		
N2	0.757 (0.417–1.375)	0.360			0.794 (0.433–1.455)	0.455		
NX	0.705 (0.278–1.787)	0.461			0.738 (0.289–1.885)	0.525		
Surgery at primary site								
Yes	1.000 (Reference)		1.000 (Reference)		1.000 (Reference)		1.000 (Reference)	
No	2.410 (1.389–4.181)	0.002	3.575 (1.968–6.494)	< 0.001	2.326 (1.335–4.053)	0.003	3.435 (1.883–6.268)	< 0.001
Radiation								
Yes	1.000 (Reference)				1.000 (Reference)			
No	2.061 (0.799–5.321)	0.135			2.020 (0.781–5.224)	0.147		
Chemotherapy								
Yes	1.000 (Reference)		1.000 (Reference)		1.000 (Reference)		1.000 (Reference)	
No	2.426 (1.464–4.019)	0.001	3.667 (2.108–6.377)	< 0.001	2.421 (1.449–4.045)	0.001	3.622 (2.065–6.353)	< 0.001
Tumor size								
< 3 cm	1.000 (Reference)				1.000 (Reference)			
< 5 cm	1.649 (0.588–4.621)	0.342			1.675 (0.597–4.701)	0.327		
≥ 5 cm	1.234 (0.474–3.215)	0.667			1.143 (0.434–3.011)	0.786		
Unknown	2.494 (1.032–6.024)	0.042			2.457 (1.014–5.954)	0.046		

Other races include Asian or Pacific Islander and American Indian/Alaska Native; Grade I = well differentiated, II = moderately differentiated, III = poorly differentiated, IV = undifferentiated; HR = hazard ratio; CI = confidence interval

## Discussion

Although GBC is a rare disease and the overall incidence has remained stable [2], a trend analysis revealed a recent increase in the incidence of late-stage gallbladder cancer [9]. However, the role of metastasis site on survival has not been addressed comprehensively to this day and the management for metastatic GBA patients remains to be explored. To our knowledge, this study is the first comprehensive study concerning the features and management of metastatic GBA on population level.

Based on our results, 788 (51.6%) patients had isolated liver metastases, 80 (5.2%) patients had isolated DL involvement, 45 (2.9%) patients had isolated lung metastases, 21 (1.4%) patients had isolated bone metastases, 2 (0.1%) patients had isolated brain metastases and 590 (38.7%) patients had multiorgan metastases. Liver was the most common site of metastases, which is in agreement with previous studies [13] and this may be because tumor cells spread to remote organs through the blood and the liver has the most blood vessels [13, 14].

Previous studies of the survival on GBA lacked a comprehensive evaluation on the prognostic value of site-specific metastases. Thus, in this study, we made a survival analysis of metastatic GBA patients, and the results showed that median OS and CSS for single metastatic GBA are both 4 months. Median OS and CSS for multi-organ metastatic GBA patients are 4 and 5 months, respectively. There is no statistically significant difference in survival between patients with single site versus multiple sites of metastases (P > 0.05), which is similar to the results of previous studies of pancreatic cancer [15] and intrahepatic cholangiocarcinoma [16]. What’s more, isolated lung metastases and DL involvement are associated with a significantly better prognosis than isolated bone metastases (P < 0.05).

Surgery is the only treatment for biliary tract cancer with long term survival. In cases with non-resectable ones (locally advanced, recurrent, or metastatic), the current standard of care favors systemic chemotherapy [17]. However, there is little evidence-based consensus about whether and when to use adjuvant therapy due to the limited utilization [18]. Some studies have proven that adjuvant therapy provides a survival benefit in node-positive or ≥ T2 disease according to the NCCN guidelines [19–22]. Some recommended chemotherapy for stage IV GBA patients with gemcitabine and cisplatin. Nevertheless, clinical response rates to these regimens are low, with < 10% long term survival and a complete response only in exceptional cases [23]. In our study, chemotherapy was associated with better OS and CSS for metastatic GBA patients. Adjuvant radiotherapy after R0 resection of GBA can improve the overall survival time and reduce the local recurrence rate [20]. A retrospective study based on National Cancer Database indicated that for unresectable but non metastatic GBA, radiotherapy combined with chemotherapy can improve survival time than using chemotherapy alone [24]. However, this conclusion may need to be verified by further prospective studies. For metastatic GBA, there is no relevant literature suggesting a survival benefit from radiotherapy. The multivariate analysis in our study indicates that radiotherapy is not related to the prognosis of metastatic GBA. Due to the advanced diagnosis and limited choices of adjuvant therapy, researchers have started to find other antineoplastic treatments. Recently, studies on targeted therapy have pointed out that there are many potential mutations in biliary tract cancer such as mutations of P53 [25], HER2 [26] and other molecular vulnerabilities, which can be used as therapeutic targets. Although there was a lack of consensus-based evidence for this new therapeutic strategy [27], researchers recommended that it is still of therapeutic significance to conduct a comprehensive genomic profiling of the tumor to identify potentially targetable aberrations and match with appropriate agent [28]. Recent immunotherapy has opened up new therapy avenues in biliary tract cancers with pembrolizumab (the PD-1 inhibitor) approved for either microsatellite instability high (MSI-H) or DNA mismatch repair deficient (dMMR) advanced solid tumors [29]. However, the rate of patients who are MSI-H and dMMR is < 5% of all biliary tract cancer patients [25]. So far, strategies incorporating immunotherapy into the treatment of patients with microsatellite stable advanced biliary tract cancers have showed largely disappointing results [29]. Thus, routine use of checkpoint inhibitors outside of clinical trials should not be recommended [28]. Because of the relative rarity and heterogeneity of GBA subtypes [30], there are few randomized prospective studies to determine the optimal treatment strategy for patients with advanced stage. Although targeted therapy and immunotherapy are in the exploratory stage, the identification of new targets and the development of new molecules are likely to make "Precision Medicine" a newly promising treatment for patients with advanced GBA.

Recently, some studies indicated that it is associated with improved survival outcomes to perform surgery at the primary site for the treatment of metastatic renal cell carcinoma and pancreas cancer [31–33]. Since the benefit from surgery was not clear for metastatic GBA patients. We use SEER database to explore the outcomes. For metastatic GBA, which was considered unresectable, patients tend to receive palliative surgery according to the current literature [7]. In our study, surgery at primary site improves median survival when tumor spreads to liver or DL. We assume there are cases of patients with metastatic indolent tumor that might be considered for resection and we propose that surgery at primary site may be a choice in certain highly selected patients with liver or DL metastases. However, methods for differentiating them from patients who are not qualified for surgery are needed to further explore. When the cancer spreads to the bone or lungs, it is not helpful to perform surgery.

Multivariate analyses of the entire cohort patients, isolated liver and DL metastasis patients all suggested that performing surgery at primary site, receiving chemotherapy were associated with better OS and CSS. Differences compared to the results of a previous study were that sex, age and marital status did not play roles in survival outcomes [2]. We suspect that this finding may be because the advanced cancer was so malignant that it eliminated differences. Although marital status was not a significant predictor for prognosis of GBA patients, interestingly, in a recent study, Dr. Joan DelFattore noted that unmarried patients may be denied potentially lifesaving treatment without objective assessment of their capacity to handle it due to the stereotype that they lack social support, which caused the high mortality of unmarried cancer patients [34].

Analyzing the prognostic consequences of metastatic GBA helps us to treat this disease in an outlook view and encourages us to apply systemic therapy. Meanwhile, we believe our results could properly counsel patients and their family about the oncologic outcomes. However, the inherent difficulties in retrospective studies of SEER database remind us to interpret the results cautiously. First, the database lacks information about comorbidities, patients who underwent surgical treatment may have better health conditions and fewer comorbidities. Second, the number of patients who underwent surgery with certain sites was not large enough. Moreover, the information of infiltrations of the liver hilum and pedicle and of the surrounding peritoneum as well as some adjacent sites of metastases such as the stomach, duodenum, pancreas, etc. was not included in the SEER database, which may also be a factor influencing the results. Despite these difficulties, the results are still convincing due to the large sample. Further prospective controlled studies to identify the highly selected subset of patients who may benefit from local treatment of the primary tumor are needed.

## Conclusion

The study showed that different metastatic sites affect survival outcomes in metastatic GBA patients. Surgery at primary site might benefit for highly selected subset of patients with liver or DL metastases. However, further prospective controlled studies to identify the highly selected subset of patients who may benefit from local treatment of the primary tumor are needed.

## Data Availability

We received permission from the National Cancer Institute, US to access the research data file in the SEER program (Accession number 10013-Nov2019). The datasets analyzed during the current study are available in the SEER repository (https://seer.cancer.gov/). The datasets used and/or analysed during the current study are also available from the corresponding author on reasonable request.

## Abbreviations

AJCC: American Joint Committee on Cancer
CI: Confidence interval
CSS: Cancer specific survival
DL: Distant lymph node
GBC: Gallbladder cancer
GBA: Gallbladder adenocarcinoma
HR: Hazard ratio
NCCN: National Comprehensive Cancer Network
OS: Overall survival
SEER: Surveillance, Epidemiology, and End Results
